# Impact of maternal whole-cell or acellular pertussis primary immunization on neonatal immune response

**DOI:** 10.3389/fimmu.2023.1192119

**Published:** 2023-06-26

**Authors:** Pablo Martin Aispuro, Daniela Bottero, María Eugenia Zurita, María Emilia Gaillard, Daniela Flavia Hozbor

**Affiliations:** Laboratorio VacSal, Instituto de Biotecnología y Biología Molecular (IBBM), Facultad de Ciencias Exactas, Universidad Nacional de La Plata, CONICET, La Plata, Argentina

**Keywords:** Pertussis, *Bordetella pertussis*, pregnancy, neonatal dose, aP, wP, priming, protection

## Abstract

With the introduction of pertussis immunization for pregnant women in many countries, there has been renewed interest in the impact of whole-cell pertussis vaccine (wP) versus acellular vaccine (aP) on disease control, particularly regarding the best approach for priming. To gather evidence on this topic, we analyzed the impact of aP or wP priming on aP vaccination during pregnancy (aPpreg) in mice. Two-mother vaccination schemes were employed (wP-wP-aPpreg and aP-aP-aPpreg), and the immune response in the mothers and their offspring, as well as the protection of the offspring against *Bordetella pertussis* challenge, were assessed. Pertussis toxin (PTx)-specific IgG responses were detected in mothers after both the second and third doses, with higher titers after the third dose, regardless of the vaccination schedule. However, a significant reduction in PTx-IgG levels was observed after 22 weeks post aPpreg immunization in mothers with the aP-aP-aPpreg scheme but not in the wP-wP-aPpreg immunized mothers. The aP-aP-aPpreg schedule triggered a murine antibody response mainly to a Th2-profile, while wP-wP-aPpreg induced a Th1/Th2 mixed profile. Both immunization schemes administered to the mothers protected the offspring against pertussis, but the wP-wP-aPpreg vaccination conferred offspring protection in all pregnancies at least up to 20 weeks after receiving the aPpreg-dose. In contrast, the immunity induced by aP-aP-aPpreg began to decline in births that occurred 18 weeks after receiving the aPpreg dose. For the aP-aP-aPpreg scheme, pups born from gestations furthest from aPpreg (+22 weeks) had lower PTx-specific IgG levels than those born closer to the application of the dose during pregnancy. In contrast, for pups born to wP-wP-aPpreg vaccinated mothers, the PTx-specific IgG levels were maintained over time, even for those born at the longest time studied (+22 weeks). It is noteworthy that only the pups born from mothers with aP-aP-aPpreg and receiving a neonatal dose of either aP or wP were more susceptible to *B. pertussis* infection than mice with only maternal immunity, suggesting interference with the induced immunity (p<0.05). However, it should be noted that mice with maternal immunity, whether vaccinated or not with neonatal doses, are better protected against colonization with *B. pertussis* than mice without maternal immunity but vaccinated with aP or wP.

## Introduction

One of the greatest public health challenges of the 21st century remains the prevention and treatment of bacterial infections (1). Although the use of antibiotics has marked a milestone in the treatment of infectious diseases, vaccination has certainly been one of the most successful health interventions in terms of lives saved and diseases prevented (2). The need of more and better vaccines and vaccination strategies to prevent diseases at different stages of life is increasingly evident in the face of the alarming increase in antimicrobial resistance and the emergence and resurgence of diseases (3). One of the most vulnerable periods of life for contracting infectious diseases that can be fatal for individuals is the first month of life (4). In the case of newborns, their immune system is not adequately developed to fight infections (5, 6). In particular, newborns exhibit deficiencies in both the quantity and quality of neutrophils, which are essential components of the innate immune system responsible for pathogen destruction during infection (7, 8). Moreover, the neonatal neutrophils express low levels of both L-selectin on the cell surface and Mac-1 (CD11b/CD18), which causes a 50% reduction in the transmigration of these cells to infection sites, and TLR4, with the concomitant deficiency in signaling *via* the MyD88 pathways. Furthermore, neonatal circulating monocytes express reduced levels of MHC class II molecules contributing to impaired APC activity (9). Intrinsic defects in B- and T-cell development and function also contributes to the diminished immune response in neonates and infants (7). Due to the unique characteristics of the neonatal immune system, newborns may require hospitalization, including stays in the neonatal intensive care unit, when they become ill, in order to facilitate their recovery. Premature or otherwise immune-compromised babies are at even higher risk of developing severe disease from a bacterium or virus that may only cause a mild disease in an older child (10). Unfortunately, even older infants can get sick and die from diseases that are preventable through vaccination because they are not old enough to receive the necessary vaccine doses that would provide them with protection. Under this context and in an effort to reverse this serious health problem in newborns and infants, vaccination during pregnancy started to be implemented in many countries as a possible solution for various pathogens (11–15). This strategy seeks to protect newborns, not only by preventing the disease in their mother, who is their main source of infection, but also by passively transferring specific immunity. The vaccination of pregnant women against pertussis, a respiratory disease, has been successfully implemented in numerous countries (16). For many years, this disease caused by the gram-negative bacterium *Bordetella pertussis* has been controlled through mass vaccination of infants. However, in recent decades, there has been a resurgence of the disease (17–20). Although the causes of its resurgence are still under debate, there is a consensus that the loss of immunity conferred by vaccination, particularly with subunit-based vaccines (also known as acellular vaccines or aP vaccines), and the prevalence of bacteria that are more resistant to the immunity conferred by aP vaccines (bacteria deficient in the expression of the immunogen called pertactin), are the most relevant causes contributing to the current pertussis epidemiology (21–25). The resurgence of the disease primarily affects newborns and infants with partial or no immunity, leading to the recommendation in 2011 by the Advisory Committee on Immunization Practices (ACIP) to administer aP boosters to all unvaccinated pregnant women (26). This aP booster is administered with a pertussis vaccines containing lower quantities of pertussis toxin (PTx) and diphtheria toxoid (Tdap, Tetanus, diphtheria, and acellular pertussis vaccine) than the pediatric DTaP vaccine (Diphtheria, Tetanus, and acellular Pertussis vaccine). The Tdap vaccine is approved for use in individuals older than 7 years of age.

To control the disease currently there are two types of vaccines in use: the first generation of pertussis vaccines consists of a suspension of detoxified and heat-killed bacteria (wP), and the second generation of vaccines made with purified *B. pertussis* immunogens (aP). The use of wP vaccines is recommended up to 7 years of age. The acellular vaccines DTaP and Tdap can be used in the pediatric population or in individuals over 7 years of age, respectively. Both formulations can be combined with other antigens, such as *Haemophilus influenzae* serotype b (Hib) or inactivated polio (IPV) and are formulated for the pediatric population.

In 2012, the recommendation for pregnancy vaccination was updated to address vaccination of all women during the third trimester of pregnancy, irrespective of their prior Tdap vaccination status, meaning vaccination in every pregnancy (Centers for Disease Control and Prevention, 2013) (27). Although the strategy has not been universally accepted to date, several Ministries/Secretaries of Health have adopted the recommendation since the strategy has been found to be safe for pregnant women and developing fetuses and newborns, as well as highly effective in preventing pertussis in infants younger than 3 months, in whom the primary vaccination series has not yet been completed (28–32). As various countries adopt the strategy of vaccination during pregnancy, increasing evidence is being obtained regarding its beneficial impact on protecting infants against pertussis (32–35). A recent systematic review has demonstrated that immunizing pregnant women against pertussis can prevent 70-90% of pertussis cases and up to 90.5% of pertussis-related hospitalizations in infants under 3 months of age (36). However, some reports have shown that administering Tdap immunization during pregnancy can lead to a decrease in humoral immune responses to subsequent immunizations using acellular pertussis antigen-containing vaccines in infants. Specifically, lower levels of anti-PTx IgG were found in infants born to Tdap-vaccinated pregnant women after the completion of primary immunization, while less consistent results were obtained following booster immunization (11, 37–39). This interference, combined with the lack of a universal schedule that includes the same type of pertussis vaccines, has sparked a debate about the advisability of schedules that include a booster during pregnancy, but with a primary vaccination schedule using wP as opposed to one using aP vaccine. In fact, questions such as the following have arisen: Does the primary vaccination scheme during childhood impact the immune response induced by the aP booster administered during pregnancy? Does the primary series using wP impact vaccination-induced immunity in pregnant women in the same way as aP priming? Do mothers vaccinated during pregnancy transfer different immunity profiles to their fetus depending on the primary vaccination scheme received? Does maternal immunity differentially interfere with infancy vaccination depending on the type of pediatric vaccine received? Finally, how long does it take for the pregnancy dose to significantly diminish the transfer of immunity to the infant?

To generate evidence in this regard and determine the potential impact of aP and wP priming on the immunity induced by the aP dose administered during pregnancy (aPpreg), we utilized the intranasal challenge mouse model. We immunized female mice using a primary schedule of 2 doses of wP or aP, along with an additional aPpreg dose, to evaluate two different vaccination schedules: wP-wP-aPpreg and aP-aP-aPpreg. We conducted comparative assessments of the immunogenicity of these regimens in both the mothers and the newborns. Furthermore, we assessed the protection against *B. pertussis* challenge in offspring born at various time points following the administration of the aPpreg dose, as well as the levels of specific antibodies in animals vaccinated or not vaccinated with a neonatal dose of aP or wP. Additionally, we performed comparisons with pups born to non-immunized mothers.

## Materials and methods

### Mice

BALB/c mice (4 weeks old), obtained from the Faculty of Veterinary Sciences, La Plata, Argentina, were kept in ventilated cages and housed under standardized conditions with regulated daylight, humidity, and temperature. The animals received food and water ad libitum. Day 1 of gestation was determined when vaginal plug was observed. Breeding cages were checked daily for new births, and the pups were kept with their mothers until weaning at the age of 4 weeks. The animal experiments were authorized by the Ethical Committee for Animal Experiments of the Faculty of Science at La Plata National University (approval number 004-06-15, 003-06-15 extended its validity until August 10, 2027).

### *B. pertussis* strain and growth conditions

*B. pertussis* Tohama phase I strain CIP 8132 was used throughout this study as the strain for challenge in the murine model of protection. Bacteria were grown in Bordet–Gengou agar supplemented with 10% (v/v) defibrinated sheep blood (BG-blood agar) for 72h at 36.5 °C. Isolated colonies were replated in the same medium for 24h and then resuspended in phosphate-buffered saline (PBS: 123 mM NaCl, 22.2 mM Na_2_HPO_4_, 5.6 mM KH_2_PO_4_ in MilliQ^®^ nanopure water; pH 7.4). The optical density at 650 nm was measured and serial 10-fold dilutions plated onto BG-blood agar to determine the number of bacteria in the challenge inoculum.

### Vaccines

The maternal immunization protocols were performed with the three-valent pertussis aP BOOSTRIX® (GSK, GlaxoSmithKline), whose composition per human dose is: pertussis toxoid (8 µg), pertactin (2.5 µg), filamentous hemagglutinin (8 µg), tetanic toxoid (20 IU), and diphtheria toxoid (2 IU) or wP pertussis vaccine from Serum Institute of India PVT LTD which composition per human dose is: Diphtheria Toxoid ≤ 25 Lf (≥ 30 IU), Tetanus Toxoid ≥ 5 Lf (≥ 40 IU), *B. pertussis* ≤ 16 OU (≥ 4 IU) adsorbed on aluminum phosphate ≤ 1.25 mg. For all experiments, immunization was carried out through the use of a 1/10 human dose of that vaccine, hereafter referred to as a mouse dose (MD). The vaccinations of neonates or infant mice were performed with 1 MD of the aP or commercial wP vaccine.

### Experimental protocol

#### Maternal and neonatal immunization

For the immunization of the mother and newborn puppies, we used the protocol previously published by our group (40). Briefly, female BALB/c mice (n=8) were intramuscularly vaccinated with 2 doses of aP or wP at days 0 and 14 plus a booster dose of aP during pregnancy (aPpreg). Before applying the third vaccine dose the females mice were housed with males within the same cage and daily checked for pregnancy, when mucosal vaginal plug was detected a third vaccine dose was applied. Mice couples stayed cohoused until the end of the experiment. Vaccination schedules tested were designated aP-aP-aPpreg and wP-wP-aPpreg. Non-immunized mice were used as negative control of protection. As we previously described (40) offspring born to either immunized or non-immunized mothers were immunized subcutaneously in the upper back with 1 MD of aP or wP at the age of 1 week (neonates). The subcutaneous route was chosen for neonatal immunization due to its practicality, considering the small size of the animals. This decision was supported by a previous unpublished study demonstrating comparable protective capacity against pertussis between the subcutaneous and intramuscular routes.

#### Protection against colonization

To evaluate the protective capacity induced by the different vaccination strategies used, intranasal challenge with a sublethal dose (10^6^–10^8^ CFU 40μl^−1^) of *B. pertussis* Tohama phase I at 14 days after the last immunization was performed as previously described (41, 42). Seven days after challenge, mice were sacrificed, and their lungs were harvested, homogenized in PBS and plated in serial dilutions onto BG-blood agar to count CFUs after incubation at 37°C for three to four days. At least three independent assays were performed.

#### Effect of neonatal vaccination on protection in mice vaccinated at birth and born to vaccinated mothers

To study the effect of active immunization of infant mice born to vaccinated mothers on protection from subsequent pertussis infection, the offspring were immunized at 7 days of age with an MD of the commercial aP or a commercial wP vaccine. Nonimmunized offspring from immunized mothers or immunized mice at 7 days of age were used as controls. Mice were challenged with *B. pertussis* 2 weeks after receiving the vaccine dose and protection assessed on day 7 as described above.

### Enzyme-linked immunosorbent assay

As we previously described (40), plates (Nunc A/S, Roskilde, Denmark) were coated with the purified pertussis toxin PTx at 3 µg/ml in 0.5 M carbonate buffer pH 9.5, by means of an overnight incubation at 4 °C. Blocked plates with 3% milk in PBS (2h 37°C) were incubated with serially diluted samples of mouse serum (1h 37°C). Serum from different periods was obtained from mice by collecting blood from the submandibular vein. The blood was allowed to clot for 1 hour at 37°C and then centrifuged for 10 minutes at 6,000xg. IgGs from individual serum or pooled sera bound to the plates were detected after a 2-h incubation with goat anti–mouse-IgG–linked horseradish peroxidase (1:8,000 Invitrogen, USA). For measuring IgG isotypes, detection of bound antibody was determined using HRP labeled subclass-specific anti-mouse IgG1 (1:8,000) or IgG2a (1:1,000) (Sigma, Aldrich). As substrate 1.0 mg/ml o-phenylendiamine (OPD, Bio Basic Canada Inc) in 0.1 M citrate-phosphate buffer, pH 5.0 containing 0.1% hydrogen peroxide was used. Optical densities (ODs) were measured with Titertek Multiskan Model 340 microplate reader (ICN, USA) at 490 nm (43, 44). From the experimental protocol performed in triplicate, one representative experiment is presented in the Results.

### Avidity assay

Avidity was measured by an ELISA elution assay as the overall strength of binding between antibody and antigen, using plates incubated for 15 min with increasing concentration of ammonium thiocyanate (NH_4_SCN) from 0 to 0.375 M. As we previously described (40), the antibody avidity was defined as the amount (percentage) of antibody retained for each increment of NH_4_SCN concentration.

### Ag-specific IFN-γ production by spleen cells

Spleens from untreated and immunized mice were passed through a 40-mm cell strainer to obtain a single-cell suspension. Spleen cells (45) were seeded in 48 well culture plates in a final volume of 500 µl/well RPMI 1640 with 10% fetal bovine serum, containing 100IU/ml penicillin and 100µg/ml streptomycin. All cell samples were stimulated with heat killed *B. pertussis* suspension (10^6^ UFC/ml), with the purified pertussis toxin PTx (2 µg/ml) or medium only. After 72 h of incubation (37°C and 5% CO2), IFN-γ concentration was quantified in supernatants by ELISA.

### Protein assay

The protein content was estimated by the Bradford method with BSA as a standard (46).

### Sodium dodecylsulfate-polyacrylamide gel electrophoresis and immunoblotting

Purified PTx solubilized in Laemmli sample buffer (47) was separated by SDS-PAGE and then transferred onto PVDF (Immobilon P, Millipore). After the transfer, PVDF membranes were probed with immune sera or non-immune sera (1:1,000) followed by incubation with anti (mouse-IgG) conjugated with alkaline phosphatase at a 1:1,000 dilution. Nitroblue tetrazolium and 5-bromo-4-chloro-3-indolyl-phosphate were used as the phosphatase substrates according to the manufacturer’s protocol (Biodynamics SRL Buenos Aires Argentina).

### Statistical analysis

The data were evaluated statistically by two-way or one-way analysis of variance (ANOVA) followed by Tukey or Šidák for multiple comparisons (via the GraphPad Prism^®^ software). Differences were considered significant at a p <0.05.

## Results

### Immunogenicity of aP-aP-aPpreg and wP-wP-aPpreg schedules in mice mothers and neonates

**Figure 1:**
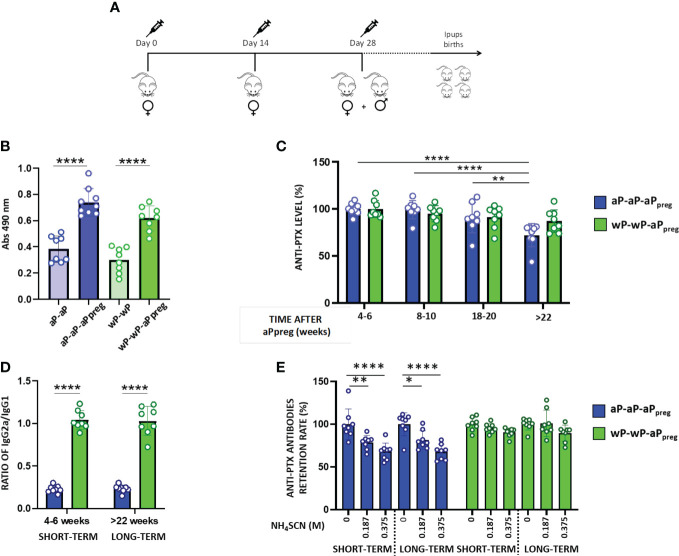
Humoral immune response in mothers primed with aP or wP. **(A)** Schematic representation of mother’s vaccination schedule. Female BALB/c mice (n=8) were intramuscularly vaccinated with 2 doses of aP or wP at days 0 and 14 plus a booster dose of aP during pregnancy (aPpreg). 1/10 of the human dose was used for each immunization. **(B)** PTx- specific IgG levels induced by 2 or 3 doses schedules in mice. Serum collected on Day 14 was analyzed by ELISA to determine the levels (absorbance values at 490 nm) of antigen PTx- specific IgG. **(C)** PTx-specific IgG levels induced by 3-dose schedules from **(A)** evaluated at different time points post-third dose. The levels of PTx-specific IgG were expressed as a percentage of the levels detected at 4-6 weeks after the third dose (considered 100%). **(D)** PTx-specific IgG2a/IgG1 ratio detected in aP- or wP-primed mothers at two time points: 4-6 weeks after receiving the aPpreg dose (short time), and 22 weeks after their last vaccine dose (long time). **(E)** Avidity of the PTx-specific IgG induced by the 2 vaccination schedules analyzed. The avidity of the PTx-specific IgG from short and long term was measured by ammonium thiocyanate (NH_4_SCN) elution and is expressed as PTx-specific antibodies retention rate (%): the percentages of PTx-specific antibodies retained after treatment with increasing concentrations of NH_4_SCN relative to that measured in absence of ammonium thiocyanate. *p<0.05, **p<0.01, ****p<0.0001 by one way ANOVA using Tukey or Šidák for multiple comparisons.

To evaluate the priming effect of the two types of vaccines used against pertussis, aP or wP, on the immune response induced by the dose applied during pregnancy (aPpreg), we administered a three-dose regimen of aP-aP-aPpreg or wP-wP-aPpreg to female Balb/C mice aged 6 to 8 weeks (**Figure 1A**). Based on the knowledge of the protective capacity of pertussis toxin and its antibodies against the disease (48, 49), the levels of PTx-specific antibodies, including IgG, IgG1, and IgG2a, were evaluated in both mothers and their offspring after receiving the vaccination schedules studied here. Non-immunized animals with the same age serve as the negative-control mice. **Figure 1B** shows that all immunized mothers induced a PTx-specific IgG response after both the second and third doses, with titers being higher after the third dose, regardless of the vaccination schedule tested. For non-immunized animals the levels of antibodies were undetectable (not shown). For the aP-aP-aPreg schedule significant differences were detected in the PTx-specific IgG levels over time after the third dose (aPpreg). For this schedule, PTx-specific IgG levels were reduced by 18% to 30% at week 22 after the third dose compared to values determined at earlier times post the third dose (p<0.0001). In contrast, for the wP-wP-aPpreg scheme, PTx-specific IgG levels remained consistent across the different times tested after the aPpreg dose (**Figure 1C**). When comparing the percentage decrease in PTx-specific IgG levels to the values detected closest to the administration of the aPpreg dose for the two vaccination schedules studied here, we observed a significant difference (p<0.0426) only in the sera obtained after week 22 of aPpreg administration. The decrease in IgG levels (as percentages) was higher in sera from mothers with the aP-aP-aPpreg scheme (27.94 ± 4.426) compared to the sera obtained from mothers vaccinated with the wP-wP-aPpreg schedule (12.92 ± 4.246).

It is important to highlight that the aP-aP-aPpreg schedule triggered murine antibody responses with the lowest IgG2a/IgG1 ratio (**Figure 1D**). The IgG2a/IgG1 ratio detected for the treatment aP-aP-aPpreg suggests that this schedule skewed, as expected, the immune response mainly to a Th2 profile. In contrast, the IgG2a/IgG1 ratio detected in wP-wP-aPpreg -immunized animals suggests that this vaccination schedule skewed the immune response mainly to a Th1/Th2 mixed profile. We also observed that mothers immunized with the wP-wP-aPpreg scheme had PTx-specific IgG with higher avidity compared to those detected in immunized mother with the aP-aP-aPpreg schedule (**Figure 1E**). This increased avidity was detected in sera obtained both shortly after the aPpreg administration (4-6 weeks) and at a later time point (+22 weeks) following pregnancy vaccination.

**Figure 2:**
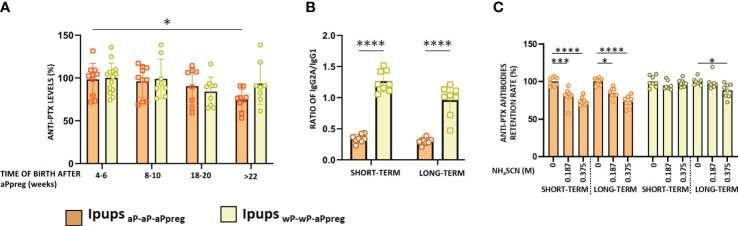
PTx-specific IgG in pups born to immunized mothers (Ipups) at different times after aPpreg. **(A)** PTx-specific IgG levels were measured at 21 days of life by ELISA. The IgG levels are expressed as the percentage relative to the level measured at 4-6weeks from each group. **(B)** PTx-specific IgG2a/IgG1 ratio detected for pups born to aP- or wP- primed mothers 4-6 weeks after the aPpreg vaccine dose (short term) and beyond 22 weeks after last vaccine dose (long term). The IgG1 and IgG2a isotypes were measured at 21 days of life in the Ipups. **(C)** Avidity of the PTx-specific IgG of the pups born to immunized mother. The avidity of the IgG antibodies obtained at 21 days of life was measured using different concentrations of NH_4_SCN and expressed as PTx-specific antibodies retention rate (%): the percentages of PTx-specific antibodies retained after treatment with increasing concentrations of NH_4_SCN relative to that measured in absence of ammonium thiocyanate. *p<0.05, ***p<0.001, ****p<0.0001 by one way ANOVA using Tukey or Šidák for multiple comparisons.

We observed that offspring born at different time points after the administration of the aPpreg dose, from mothers who received different vaccine schedules (hereafter designated as either Ipups_wP-wP-aPpreg_ or Ipups_aP-aP-aPpreg_), exhibited PTx-specific antibody responses (**Figure 2**). In the case of the aP-aP-aPpreg scheme, PTx-specific IgG levels were lowest in pups born from gestations furthest from the aPpreg administration (+22weeks), in comparison to those born closer to the dose application during pregnancy (**Figure 2A**). In contrast, for Ipups_wP-wP-aPpreg_ the PTx-specific IgG levels were maintained over time of birth, even for those born at the longest time studied here (+22weeks). We also observed differences in the IgG2a/IgG1 ratio between Ipups_wP-wP-aPpre_ and Ipups_aP-aP-aPpreg_. The former exhibited a ratio of IgG2a to IgG1 greater than 1, indicating a predominantly Th1-type response profile, whereas the latter showed a ratio of less than 1, suggesting an immune response skewed towards a profile mainly of the Th2 type (**Figure 2B**). An interesting finding was the detection of higher avidity of anti-PTx IgG in the sera of mice born to mothers immunized with the wP-wP-aPpreg scheme, in comparison to those induced in mice born to mothers immunized with the aP-aP-aPpreg schedule (**Figure 2C**). Point-to-point comparisons between the two treatments can be found in the **supplementary material (MS1)**. The enhanced affinity was observed in the sera of Ipups_wP-wP-aPpreg_ born both shortly after (4-6 weeks) and at a later time point (+22 weeks) following the maternal vaccination dose during pregnancy.

**Figure 3:**
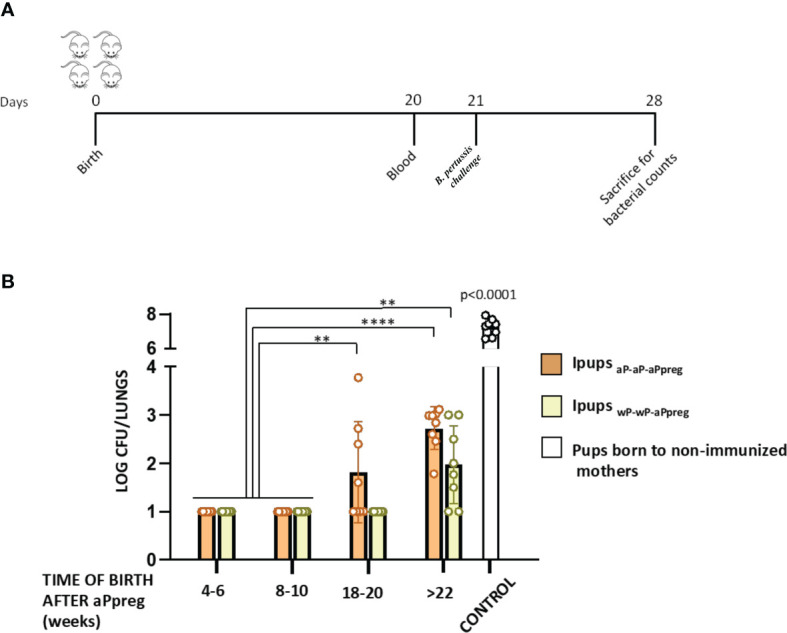
Effect of maternal immunization on protection of offspring against *Bordetella pertussis* infection. **(A)** Mice (n= 7) born between 4-6 to beyond 22 weeks after the aPpreg vaccination dose to aP- or wP primed mothers or non-immunized females (controls) were challenged with a sublethal dose (5x10^6 40 μl^-1^) *B. pertussis* Tohama phase I at 21 days after birth followed by sacrifice 7 days after challenge. **(B)** The number of bacteria recovered from mouse lungs, expressed as the log_10_ of CFUs per lungs, is plotted on the ordinate, time when Ipups were born after aPpreg immunization is indicated in weeks on the abscissa, with the data representing the means ± the SD. CFUs recovered from pups born to non-immunized female mice were also presented. The dotted horizontal line indicates the lower limit of detection. **p<0.01, **** p<0.0001, by two way ANOVA using Šidák for multiple comparisons.

According to our previous findings on the aP-aP-aPpreg schedule (40), we observed that the protective capacity against *B. pertussis* infection (**Figure 3A**) remained almost intact in offspring born before 18 weeks after receiving the aPpreg dose (**Figure 3B**). However, in offspring born beyond 22 weeks after their mothers received the aPpreg dose, we detected a reduction in protection (**Figure 3B**). Specifically, we found that the level of recovered CFUs from the lungs of those pups increased by over 1.7 orders of magnitude compared to levels detected in offspring born closer to the time of aPpreg administration (p<0.0001). Notably, we observed that the loss of protective capacity detected in Ipups_aP-aP-aPpreg_ was not seen in offspring born to mothers vaccinated with the wP-wP-aPpreg scheme. Only a slight loss of protective immunity was observed in pups born 22 weeks (not earlier, as in the case of pups with maternal immunity induced by the aP-aP-aPpreg scheme) after the wP-wP vaccinated dam received the aPpreg dose (p<0.001, **Figure 3B**). We also observed that Ipups born at 18-20 weeks and >22 weeks from wP-wP-aPpreg dams exhibited a higher level of protection against *B. pertussis* lung colonization compared to pups born at the same time from aP-aP-aPpreg dams (with p<0.01 and p<0.05, respectively). As expected, pups born from non-immunized mothers, which were used as controls in these assays, exhibited the highest levels of *B. pertussis* colonization (log recovered CFUs/lungs around 7.2, **Figure 3B**).

### Neonatal immunization in pups born to immunized mothers with different primary schedules

**Figure 4:**
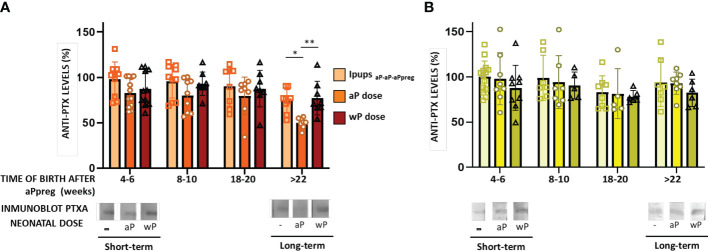
Humoral immune response in neonates born to immunized mothers with different primary schedules and immunized with aP or wP vaccine at 7 days of life. The Pups born to immunized mothers, Ipups_wP-wP-aPpreg_ or Ipups_aP-aP-aPpreg_, were immunized at 7 days of age either with aP- or wP-vaccine. Non-immunized pups born to either vaccinated or naïve mothers were used as the controls group. PTx-specific IgG levels were measured in the serum of mice 21 days of age who were born to mothers primed with either aP **(A)** or wP vaccine **(B)**, at various time points after aPpreg. Specific anti-PTx IgG titers are expressed as a percentage of the IgG levels determined in non-immunized pups born 4-6 weeks after maternal immunization (100%). At the bottom of the figures immunoblotting of purified PTx separated by 12.5% (w/v) SDS-PAGE and probed with the polyclonal antisera obtained from Ipups immunized either with aP- **(A)** or wP-vaccine **(B)** were presented. The avidity of the IgG antibodies was also measured in Ipups_aP-aP-aPpreg_ **(C)** or Ipups_wP-wP-aPpreg_ **(D)** at 21 days of life and born at short term and long term after aPpreg. The avidity is indicated by the percentages of PTx-specific antibodies retention rate after treatment with increasing concentrations of ammonium thiocyanate (NH_4_SCN). *p<0.05, **p<0.01, ***p<0.001 ****p<0.0001 by one way ANOVA using Šidák for multiple comparisons.

**Figure 5:**
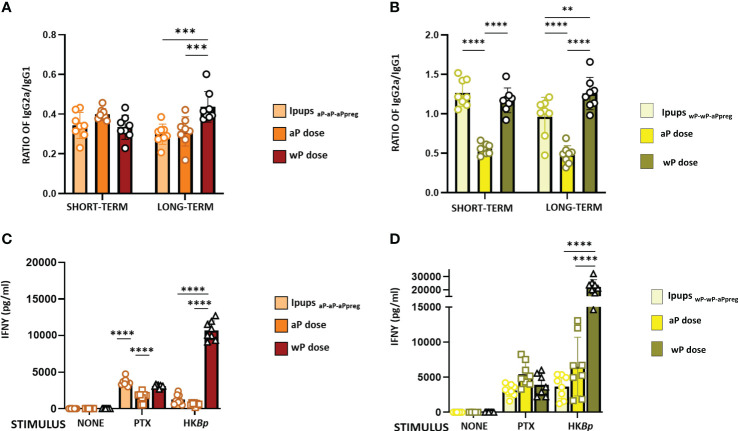
IgG2a/IgG1 and IFNγ levels in neonates born to immunized mothers with different primary schedules and immunized with aP or wP vaccine at 7 days of life. Specific anti-PTx IgG2a/IgG1 were measured in the serum of mice 21 days of age who were born to mothers primed with either aP **(A)** or wP vaccine **(B)**, at different time points after aPpreg. IFNγ levels detected in Ipups_aP-aP-aPpreg_ **(C)** or Ipups_wP-wP-aPpreg_ **(D)** born at short term after aPpreg. Thirty days after the last immunization, mice were sacrificed and their spleen cells stimulated with heat killed *B. pertussis* (HBp), purified PTX or medium alone (negative control). After 72 h of culture, the concentrations of IFNγ were determined in the culture supernatant by ELISA. The results are expressed as mean values (± SEM) n=8. **p<0.01, ***p<0.001, ****p<0.0001 by one way ANOVA using Šidák for multiple comparisons.

**Figure 6:**
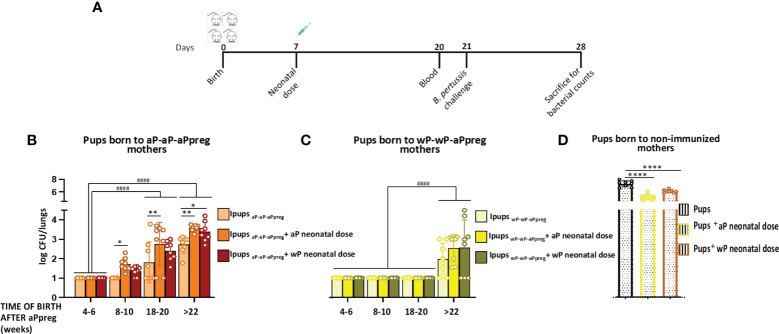
Effect of neonatal immunization on protection against *B*. *pertussis* infection of pups with maternal immunity. **(A)** Schematic representation of vaccination and challenge protocols. Neonatal mice with 7 days of age either without or with maternal immunity were vaccinated with a commercial aP, or commercial wP vaccines (n=8 in each group) were challenged with a sublethal dose (5x10^6 40 μl^-1^) *B. pertussis* Tohama phase I at 21 days after birth followed by sacrifice 7 days after challenge. **(B, C, D)** The number of bacteria recovered from mouse lungs, expressed as the log10 of CFUs per lungs, is plotted on the ordinate, time when Ipups were born after aPpreg immunization is indicated in weeks on the abscissa, with the data representing the means ± the SD. CFUs recovered from pups born to non-immunized female mice were also presented. The dotted horizontal line indicates the lower limit of detection. *p<0.05, **p<0.01, ****p<0.0001 by one way ANOVA using Šidák for multiple comparisons. #### p<0.0001 by two way ANOVA using Šidák for multiple comparisons.

We have extended our previous findings on neonatal immunization (40) by comparing the effect of this dose on transferred maternal immunity induced by two different three-dose schedules that we studied here. As in our previous study, neonatal immunization was administered at seven days of age using either an aP- or wP-vaccine. As control groups, we used pups born to immunized mothers who did not receive the neonatal dose, pups born to non-immunized mothers who did not receive the neonatal dose, and pups born to non-immunized mothers who received the neonatal dose with either an aP or wP vaccine. We assessed the immunogenicity and protective capacity induced by the neonatal dose in pups from different litters, as shown in **Figures 4**–**6**. For pups born in the short term (i.e., 4-6 weeks after their mother received the aPpreg vaccine), we observed that the immunity transferred from either aP-aP-aPpreg or wP-wP-aPpreg mothers was not affected by the neonatal dose’s induced by either aP or wP (**Figures 4A, B**). In the case of neonatal immunization with aP in Ipups_aP-aP-aPpreg_, the levels of PTx-specific IgG appeared somewhat decreased compared to those detected in the control group that did not receive the neonatal dose. However, this difference only became significant (p<0.05) in pups born longer after the aPpreg dose application (i.e., +22 weeks). This interference of the neonatal dose with the maternal immunity was observed for the aP neonatal dose but was not detected for the wP neonatal dose, as shown in **Figure 4A**. Consistent with the ELISA-IgG results obtained, a differential recognition intensity of PTx in blots from SDS-PAGE gels probed with the tested vaccine-induced sera was observed, as shown in the bottom of **Figures 4A, B**. It is noteworthy that while the PTx band was clearly recognized by the specific antibodies in mice with maternal immunity, recognition with anti-PTx antibodies was diminished in mice with maternal immunity that received a dose of aP at seven days after birth (**Figure 4A**). This reduction in PTx recognition was not observed in Ipups_wP-wP-aPpreg_ immunized with either aP or wP neonatal dose (**Figure 4B**). Regarding sera avidity, no difference was detected by ELISA assays between the sera from Ipups_aP-aP-aPpreg_ born either in the short term or long term after aPpreg administration (**Figure 4C**). For non-immunized control pups born to aP-aP-aPpreg mothers, as well as for Ipups born to aP-aP-aPpreg mothers and immunized with either aP or wP neonatal dose, we observed a decline in PTx-specific IgG retention levels when the NH_4_SCN chaotropic agent was used, regardless of the concentration used (**Figure 4C**). Contrary to what was observed for Ipups born to mothers vaccinated with the aP-aP-aPpreg regimen, we practically did not detect a decline in PTx-specific IgG retention for Ipups_wP-wP-aPpreg_ offspring, even in the case of the highest concentrations of NH_4_SCN agent (**Figure 4D**). Only a small reduction in specific IgG retention was detected in animals born long after aPpreg administration and not receiving neonatal vaccine doses. A point-to-point comparative analysis of the quality of sera obtained from offspring born to mothers who received the different vaccination schedules here studied, revealed that the avidity of sera from offspring with the aP-aP-aPpreg scheme was lower compared to sera from offspring born to mothers immunized with the wP-wP-aPpreg scheme (**Supplementary Material MS1**). Moreover, this reduced avidity observed in aP-aP-aPpreg offspring could not be reversed by neonatal immunization with either aP or wP. In contrast, the higher avidity observed in offspring born to mothers immunized with the wP-wP-aPpreg scheme persisted even in those who received the neonatal dose with aP or wP (**Supplementary Material MS1**). All these results show a better quality of antibodies for Ipups_wP-wP-aPpreg_ offspring, with or without acquired immunity from neonatal vaccination, compared to those found in Ipups born to aP-aP-aPpreg mothers.

In this study, we also evaluated the IgG1 and IgG2a isotypes in the pups born to immunized mothers (**Figure 5**). The results showed that in the case of Ipups_aP-aP-aPpreg_ either non-immunized or immunized with a neonatal dose, IgG1 levels were significantly higher than those of IgG2a (**Figure 5A**). For the Ipups_aP-aP-aPreg_ born > 22weeks after the aPpreg dose administration, the IgG2a/IgG1 ratio was highest in Ipups vaccinated with a wP neonatal dose (p<0.001). It is important to note that for Ipups_aP-aP-aPpreg_ born between weeks 18-20 after the mother received the aPpreg dose, the neonatal dose did not induce an increase of IgG1 levels (**Supplementary material MS2**), thus indicating again an interference phenomenon between maternal immunity and the aP induced immunity. Only a slight increase in IgG2a levels was observed in offspring who received the neonatal dose with aP, compared to those with only maternal immunity from the aP-aP-aPpreg scheme. For offspring born after week 22 of aP-aP-aPpreg mothers receiving the aPpreg dose, a slight increase in IgG1 or IgG2a levels was detected after the neonatal dose with aP or wP, respectively (**Supplementary material MS2**).

In Ipups_wP-wP-aPpreg_, the levels of IgG1 and IgG2a were similar (**Figure 5B**). Moreover, in these Ipups, the wP neonatal dose induced a higher Th1/Th2 profile than the aP one (see **Figures 5A, B**). These results were observed in both short- and long-term periods. In contrast to our observations in Ipups_aP-aP-aPpreg_, offspring born at weeks 18 and 22 from mothers immunized with the wP-wP-aPpreg scheme and receiving the aPpreg dose showed increased levels of IgG1 upon neonatal administration of aP or wP (**Supplementary Material MS2**). Furthermore, a significant increase in IgG2a levels was observed in offspring born at weeks 18 and 22 and immunized with a neonatal wP dose in the wP-wP-aPpreg group. Detailed comparative data can be found in **Supplementary Material MS2**.

The higher magnitude of the Th1 profile detected in Ipups_wP-wP-aPpreg_, in comparison with that of Ipups_aP-aP-aPpreg_, in which Th2 was the predominant profile, was confirmed by IFNγ determinations (**Figures 5C, D**). In the short term, the IFNγ levels in Ipups_aP-aP-aPpreg_ vaccinated with aP neonatal dose were even lower than those detected in the control group of pups with maternal immunity and without the neonatal dose (p<0.0001), suggesting interference from maternal immunity (**Figure 5C**). When heat killed *B. pertussis* was used as stimulus, the highest value of IFNγ levels was detected for the Ipups_aP-aP-aPpreg_ vaccinated with wP neonatal dose (p<0.0001). In contrast, no interference between the transferred maternal immunity and the immunity induced by any neonatal dose was detected in the Ipups_wP-wP-aPpreg_. Once again, when heat-killed *B. pertussis* was used as a stimulus, the highest value of IFNγ levels was detected in the Ipups_wP-wP-aPpreg_ vaccinated with wP neonatal dose (p<0.0001) (**Figure 5D**).

### Neonatal immunization and protection against *B. pertussis* infection in mice born to immunized mothers with different primary schedules

As we previously reported, neonatal animals born to aP-aP primed mothers who had not received vaccination and were born before week 18 following the administration of the aPpreg vaccine demonstrated a high level of protection against *B. pertussis* infection (40). The CFU counts in the lungs of pups with aP-aP-aPpreg maternal immunity were non-detectable. Moreover, all pups born 4-6 weeks after their mothers received the aP pregnancy dose and were immunized at 7 days of age were also protected, regardless of their mother’s vaccination schedule or the type of vaccine used as a neonatal dose (**Figure 6**). However, protection against *B. pertussis* infection started to decline in aP-immunized pups born later than 8 weeks after their aP-aP-aPpreg vaccinated mothers received the pregnancy aP dose (**Figure 6B**). Notably, the highest CFU values were detected in Ipups_aP-aP-aPpreg_ born after 22 weeks from the aPpreg administration and either vaccinated with aP or wP, indicating that the immunity induced by the neonatal dose interfered with maternal immunity from the aP-aP-aPreg schedules (**Figures 6B, C**). Additionally, it is important to note that the CFU levels in all pups with maternal immunity, whether vaccinated or not vaccinated with the neonatal dose, were lower than those observed in pups born to non-vaccinated mothers (**Figure 6D**). The obtained data on aP neonatal dose were consistent with the levels of anti-PTx antibodies observed, demonstrating not only their protective role but also the consequences of interference mechanisms between maternal and neonatal immunity.

In the Ipups_wP-wP-aPpreg_ group, where IgG interference between maternal and neonatal immunity was not detected, neonatal vaccination did not affect the protective capacity induced by maternal immunity. For Ipups_wP-wP-aPpreg_ mice born before week 20 after their mothers received the aPpreg dose, transferred maternal immunity was not affected by aP or wP neonatal dose (**Figure 6C**). However, for mice born after week 22, a decrease in the protective capacity conferred by maternal immunity was detected. This decrease was not overcome by neonatal vaccination. It is worth noting that the levels of CFUs recovered from the lungs in Ipups_wP-wP-aPpreg_ unimmunized or immunized with a neonatal dose of aP or wP were significantly lower (higher protection) than those detected in vaccinated or non-vaccinated Ipups_aP-aP-aPpreg_ (p<0.0001). A comparison of the bacterial burden in offspring born to mothers with different vaccination schedules after each neonatal dose was presented in the **Supplementary Material MS3**. As shown in panel A of **Figure MS3**, in offspring born 18-20 weeks after the mother with wP-wP-aPpreg received the aPpreg dose, the neonatal dose of aP or wP kept the recovered bacterial load from the lungs below the detection limit, similar to the levels detected in offspring with maternal immunity but without receiving the neonatal dose. On the contrary, the neonatal dose of aP or wP in offspring born 18-20 weeks after the mother with aP-aP-aPpreg received the aPpreg dose did not induce the same level of protection, rather, their effect led to higher colonization levels than those detected in offspring with the same neonatal doses but born to wP-wP-aP mothers. In the case of offspring born after 22 weeks of the mother receiving the aPpreg dose, the neonatal dose of aP was more effective in preventing lung colonization in offspring with wP-wP-aPpreg maternal immunity than those with aP-aP-aPpreg maternal immunity. Moreover, this difference in efficacy in protection against bacterial lung colonization between offspring with different maternal immunity was observed even in offspring that did not receive any neonatal dose (**Figure MS3b**).

## Discussion

Newborns and non-adequately or unvaccinated infants are particularly susceptible to certain infectious diseases, much more so than older children and adults. Because the potential benefits of vaccinating pregnant women extend not only to the health of the woman but also to that of her offspring, vaccination during pregnancy deserves special consideration (50, 51). In fact, many countries have implemented the vaccination of pregnant women with nonviable/inactivated vaccines such as seasonal influenza, COVID-19, and diphtheria, tetanus, and pertussis vaccines, with very good results in terms of safety and prevention of serious diseases in both the mother and her child (38, 39, 51). Immunization during pregnancy against pertussis has been extended in several countries due to an increase in pertussis cases in recent years (12, 35, 52–54). However, this strategy has not been universally adopted, partly due to unfounded fears of vaccination during pregnancy and concerns about interference with the immune response to childhood vaccination (16). The data collected so far shows that this interference has no impact on the appearance of new clinical cases (16). Adding information about this strategy will undoubtedly contribute to the decision-making process on whether to universalize this strategy or not. In this study, we utilized a murine model to evaluate the effects of primary vaccination schemes using the two commonly used vaccines (wP or aP) on the immunity generated by vaccination during pregnancy (aPpreg) in both the mother and her offspring. This analysis on the possible imprinting immune response arose because there are currently populations of women who have received a primary vaccination scheme against pertussis covered by either wP or aP. Then, during pregnancy, these women receive the same acellular vaccine, despite having a different imprinting response. The results obtained from the murine model confirmed the priming effect, which skews the immunological response towards profiles that correspond to the vaccine used in the primary series. On one hand, we detected a longer duration of transferable immunity to the pups from mothers in which the primary scheme was carried out with wP. This data is consistent with evidence showing that wP induces a longer-lasting immune response than aP (55). Therefore, we observed that in mothers vaccinated with the wP-wP-aPpreg scheme, PTx specific IgG levels only declined to less than 17% after week 22 of receiving the aPpreg dose, compared to the decline of more than 30% detected in mothers vaccinated with aP-aP-aPpreg after week 22 of receiving the aPpreg dose (**Figure 1C**). Furthermore, we observed that while PTx specific IgG levels did not vary among the wP-wP-aPpreg litters born at different times post aPpreg administration, a drop in PTx specific IgG levels was observed for aP-aP-aPreg pups born after weeks 18 of receiving the aPpreg dose (**Figure 2A**). Antibody affinity was higher in pups born to wP-wP-aPpreg mothers (**Figure 2C**). Additionally, we observed differences in the IgG2a/IgG1 ratio, which was higher in pups born to wP-wP-aPpreg mothers than in pups born to aP-aP-aPpreg mothers. These results show a bias towards the Th1 profile in the case of wP-wP-aPpreg pups, which did not change throughout the litters. Moreover, these results were confirmed with IFNγ measurements, a marker of the Th1 profile (a recommended profile to induce protection (56). The data obtained are consistent with the knowledge about the type of immune response that each of the vaccines used induces (56).

As for any vaccine, one of the continuous challenges is to maintain high vaccination coverage to achieve direct effects on individuals and the indirect effect of herd or community immunity. The COVID-19 pandemic has caused a decrease in vaccination coverage overall, and each country is working to recover it (57). In the case of vaccination during pregnancy, low coverage not only impacts the mother’s health but also that of the child. This situation, coupled with preterm births, underscores the need to complement the vaccination strategy during pregnancy with another that has a positive impact on the health of the newborn and infant. Postpartum vaccination is especially beneficial for mothers, reducing the risk of infection in the newborn by the mother. However, it is not possible to adequately transfer immunity to the newborn since its passage occurs only through lactation and 2 weeks after the mother receives the vaccination dose, leaving the newborn very vulnerable in the first few days of life (58). In this context, neonatal vaccination has once again been discussed to provide early protection, narrowing the critical period of vulnerability intrinsic to routine vaccination schedules that start later in life (59, 60). In previous studies, we analyzed whether immunization of neonatal mice with pertussis vaccines inducing different Th-profiles leads to protection against *B. pertussis* (40). Our study showed that the protection against *B. pertussis* was higher when neonatal mice were immunized with aP compared to wP. Furthermore, protection against pertussis was enhanced with a scheme of two immunizations, at 7 and 21 days of age (40). We also found that the protection induced by maternal aP-antibodies in mice from aP-aP-aPpreg mother’s schedule was not affected by neonatal immunization with aP or wP. However, we observed a slight decrease in protection in pups born at least 10 weeks after their mothers received the aP dose, specifically with neonatal immunization with aP (40). In the experiments here presented, we found that the level of protection detected in pups born after 8 weeks of the mother receiving the primary aP-aP scheme and the aPreg dose, and vaccinated at 7 days with aP, was lower compared to that detected in pups with maternal immunity but not receiving any neonatal dose. These results once again demonstrated interference of aP-induced immunity in the protection induced by maternal immunity. This loss of protection became even more evident in pups born at later times from the aPreg dose. For the pups born to aP-aP-aPpreg mothers beyond 18 weeks, we detected a loss of protective immunity compared to pups born closer to the administration of aPreg that could not be recovered with neonatal dose of aP or wP. However, it is noteworthy that the levels of protection achieved even in pups born at later times from the administration of aPreg were higher than those detected in pups immunized with neonatal doses (aP or wP) but without maternal immunity. These results on the dependence of the type of vaccines used for mothers and neonates in the interference are in agreement with those previously reported by us and by Feunou et al, who showed that protection induced by infant vaccination was affected by maternal antibodies if the vaccine used in infancy was the same than the vaccine used in pregnancy (61). The mechanisms of the interference between maternal immunity and vaccine-induced immunity during infancy are still debated. It has been observed in a murine model, however, that the induction of germinal center (GC) B cell responses occurs even when early-life antibody responses are abrogated by maternal antibodies (62). GC B cells induced in the presence of maternal antibodies form GC structures and exhibit canonical GC changes in gene expression but fail to differentiate into plasma cells and/or memory B cells in a maternal antibody titer-dependent manner. Furthermore, GC B cells elicited in the presence or absence of maternal antibodies show differences in genes associated with B cell differentiation and isotype switching. The authors of ref 65 concluded that maternal antibodies do not prevent B cell activation but control the output of the GC reaction both quantitatively and qualitatively, shaping the antigen-specific B cell repertoire.

In the context of the aP-aP-aPreg immunity, it was possible to correlate the loss of protection with the PTx specific IgG levels (blunting effect (63),). On the contrary, protection against colonization by *B. pertussis* induced by maternal immunity transferred from mothers with a wP-wP-aPpreg vaccination schedule to offspring remained at very adequate levels for the different litters born at different times after the administration of the aPpreg dose to the mothers. Therefore, even in offspring born in week 18 after the mother received the aPpreg dose, colonization by *B. pertussis* was lower than the detection limit of the counting method. Furthermore, no interference in the protection induced by maternal immunity was detected for these offspring with the administration of the neonatal dose at 7 days, either with aP or wP. Only in offspring (Ipups_wP-wP-aPpreg_) born after week 22 since their mothers received the aPpreg dose was a reduction in protection detected, both for those who did not receive the neonatal dose and for those who received aP or wP at 7 days of life. Again, the loss of protective capacity detected in the Ipups_wP-wP-aPpreg_ offspring born in week 22 after their mothers received the aPpreg dose is relatively low, as the levels of *B. pertussis* colonization detected in this case are several orders of magnitude lower than those found in mice without maternal immunity but with immunity induced by the neonatal dose of aP or wP. It should be noted that in offspring born at different times with respect to the vaccination of mothers with the wP-wP-aPpreg schedule, the quality and levels of antibodies are maintained, unlike what was detected in offspring born from mothers with the aP-aP-aPreg schedule. Furthermore, offspring born from wP-wP-aPpreg mothers are characterized by presenting an immune response with a Th1/Th2 mixed profile, with Th1 being of higher magnitude than that detected for aP-aP-aPreg offspring. The IFNγ levels detected confirmed these results.

At this point, it is important to note that the protection levels we refer to are regarding bacterial colonization in the lower respiratory tract. For pertussis, a highly contagious disease, it is also important to analyze colonization in the upper respiratory tract, as reducing it would lead to a decrease in transmission. Published data in the baboon model has shown that the aP vaccine is ineffective in clearing bacteria from the nose (64). The wP vaccines achieve a greater reduction, but improvements are necessary to significantly reduce transmission (64). Mucosal administration vaccines, such as the attenuated BPZE vaccine or others that induce mucosal immune responses, will be key to achieving this goal (21, 65, 66).

All of the presented results indicate that, in the animal model, both wP and aP vaccines have a priming effect that affects the aPpreg dose differently and corresponds to superior long-term protection against pertussis observed with wP vaccine. Acellular vaccine priming is associated with a skewing of the immune response towards a more Th2-like response, whereas whole-cell priming is associated with a Th1 response. Whole-cell priming followed by aPpreg vaccine booster results in better PTx-specific IgG avidity through the induction of an IgG2a/IgG1 mixed profile. Our results support the feasibility of a neonatal vaccination strategy against pertussis, even in infants who have received maternal immunity. However, for infants with maternal immunity induced by aP-aP-aPreg, the neonatal dose would only be recommended for infants born close to the aPreg dose. In cases of maternal immunity derived from the wP-wP-aPpreg scheme, the neonatal dose can be applied even in infants born further away from the aPpreg dose. In summary, we have demonstrated that neonatal immunization could be used in parallel with maternal vaccination strategy to increase the newborn/infant population’s immunity against pertussis. The potential blunting of protection conferred by maternal immunization through infant vaccination could depend on the type of vaccine used in infancy (priming effect) and in neonates.

## Data availability statement

The original contributions presented in the study are included in the article/**Supplementary material**. Further inquiries can be directed to the corresponding author.

## Ethics statement

The animal study was reviewed and approved by Ethical Committee for Animal Experiments of the Faculty of Science at La Plata National University.

## Author contributions

DH planned the study, made the laboratory analysis, interpreted data, and drafted manuscript. PM, DB, EG, and EZ performed certain experiments and laboratory analyses, interpreted data, and revised figures and the manuscript. All authors approved the final manuscript. All authors contributed to the article.
