# Supplementary material for: Impact of maternal whole-cell or acellular pertussis primary immunization on neonatal immune response
Source: Front Immunol. 2023 Jun 26;14:1192119. doi: 10.3389/fimmu.2023.1192119 (PMC10330814; doi:10.3389/fimmu.2023.1192119)
Supplement: Supplementary file 1 [file DataSheet_1.docx]

MS1

The immune response induced by the two types of vaccines tested in our study differs significantly, as described by multiple authors and summarized in our work. The acellular vaccines primarily stimulate a humoral response with a Th2 profile, while the whole cell vaccine elicits a mixed profiles with a strong Th1 profile. Therefore, our aim was not to directly compare the two schemes, but rather to examine how the response changes over time within each scheme.

Here we add an analysis on the percentage of decline between the different vaccination schemes used her. Specifically, we compared the results obtained at 18-20 weeks after the mother received the aPpreg dose with those obtained after 22 weeks following the administration of the aPpreg dose. In Figure 1C, we identified a significant difference (p<0.0426) only for sera obtained after week 22 of aPpreg administration. Notably, there was a greater decrease in IgG levels in the sera from aP-aP-aPpreg mothers (27.94 ± 4.426) compared to those from wP-wP-aPpreg mothers (12.92 ± 4.246).

We apologize for any confusion that may have arisen and thank the reviewer for their valuable input. We have revised the manuscript to provide clearer explanations and address any potential misunderstandings.

For Figure 1E, the comparative results were as follows.

| **SCN concentration (M)** | **IgG-PTx drop (%) in Ipups aPaPaP** | **IgG-PTx drop (%) in Ipups wPwPaP** | **Statistical significance** |
| --- | --- | --- | --- |
| **0,1875 Short term** | 21,29±2,887 | 4,498±1,878 | *** p< 0.001 |
| **0,375 Short term** | 32,09±3,604 | 9,568±1,824 | **** p< 0.0001 |
| **0,1875 Long term** | 18,34±3,813 | -1,047±5,475 | * p<0.0324 |
| **0,375 Long term** | 32,32±3,225 | 10,48±3,374 | *** p< 0.001 |

These same analyses were performed for the data presented in Figures 2A, 4A, and 4B, and no significant differences were found. However, significant differences were detected for Figures 2C, 4C, and 4D.

For the case of Figure 2C, these were the results obtained.

| **Figure 2 C:** | **IgG-PTx drop (%) in Ipups aPaPaP** | **IgG-PTx drop (%) in Ipups wPwPaP** | **Statistical significance** |
| --- | --- | --- | --- |
| **0,375 M SCN short term** | 26,08±1,868 | 1,802±1,676 | **** p<0.0001 |
| **0,375 M SCN long term** | 26,86±2,831 | 11,98±3,248 | ** p<0.01 |

These presented results show that the avidity of the sera obtained from offspring born to mothers immunized with the aP-aP-aPpreg scheme was lower compared to that observed in offspring of wP-wP-aPreg mothers. These findings were observed for offspring born both in the short term after the aPpreg dose administration and in the long term.

For Figures 4C and 4D, the results were as follows.

| **Figures 4 C y D** | **IgG-PTx drop (%) in Ipups aPaPaPreg** | **IgG-PTx drop (%) in Ipups wPwPaPpreg** | **Statistical significance** |
| --- | --- | --- | --- |
| **0,375 M SCN aP boost short term** | 28,37±1,557 | 8,704±1,898 | **** p<0.0001 |
| **0,375 M SCN aP boost long term** | 27,68±1,488 | 1,965±1,671 | **** p<0.0001 |
| **0,375 M SCN wP boost short term** | 37,53±1,544 | 7,329±2,55 | **** p<0.0001 |
| **0,375 M SCN wP boost long term** | 31,37±1,291 | 3,691±1,667 | **** p<0.0001 |

This point-to-point comparative analysis of the quality of sera obtained from offspring born to mothers who received different vaccination schedules revealed that the avidity of sera from offspring with the aP-aP-aPpreg scheme was lower compared to sera from offspring born to mothers immunized with the wP-wP-aPpreg scheme. Moreover, this reduced avidity observed in aP-aP-aPpreg offspring could not be reversed by neonatal immunization with either aP or wP. In contrast, the higher avidity observed in offspring born to mothers immunized with the wP-wP-aPpreg scheme persisted even in those who received the neonatal dose with aP or wP. All this data was included in the new version of the manuscript as Supplementary Material MS1.
