# Supplementary material for: Impact of maternal whole-cell or acellular pertussis primary immunization on neonatal immune response
Source: Front Immunol. 2023 Jun 26;14:1192119. doi: 10.3389/fimmu.2023.1192119 (PMC10330814; doi:10.3389/fimmu.2023.1192119)
Supplement: Supplementary file 2 [file Image_1.pdf]

MS2

Pups born to aP-aP-aPpreg immunized mothers

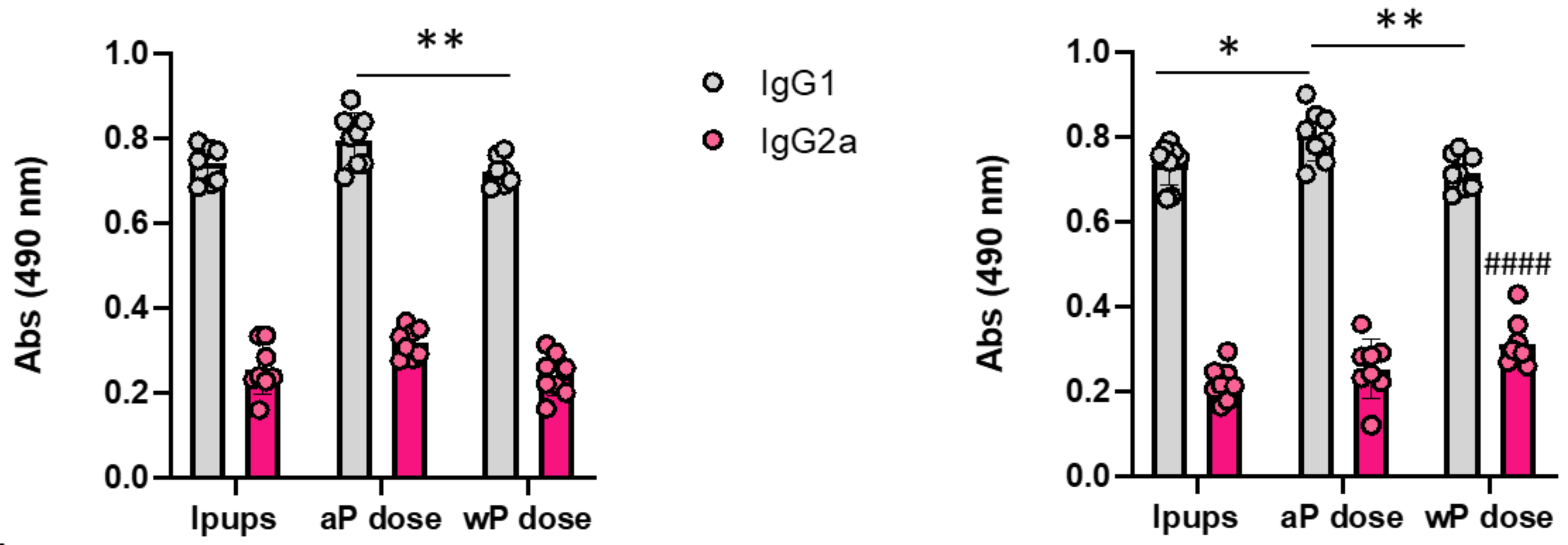

Time of birth after aPpreg (weeks) 4-6 >22

*** for IgG1 statictics**
# for IgG2a statictics

Pups born to wP-wP-aPpreg immunized mothers

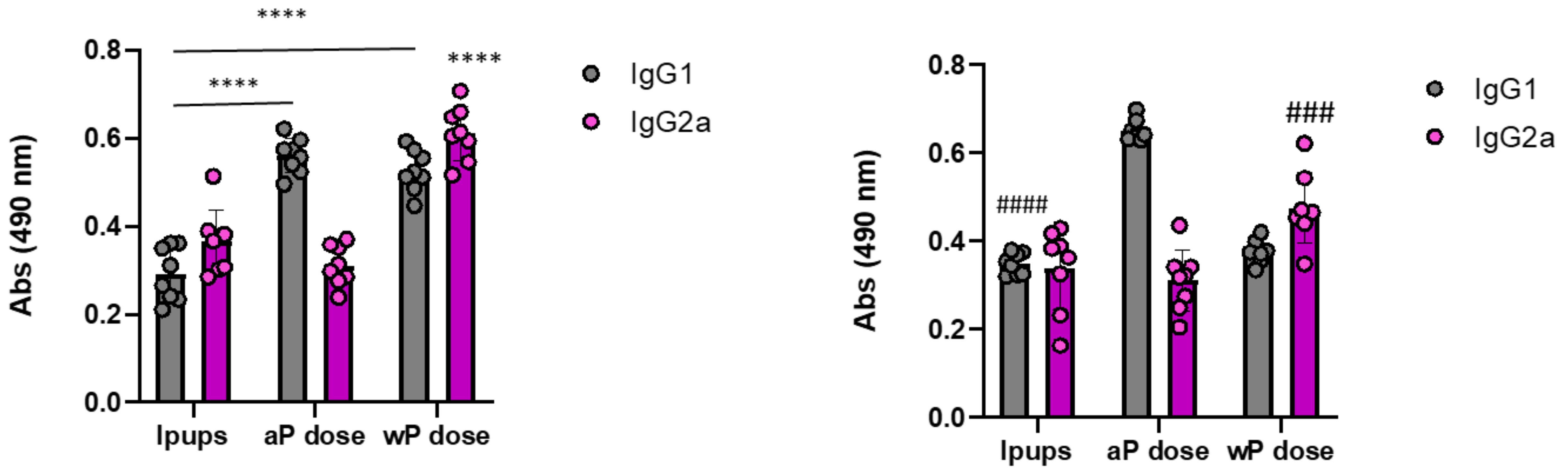

Time of birth after aPpreg (weeks) 4-6 >22

MS3

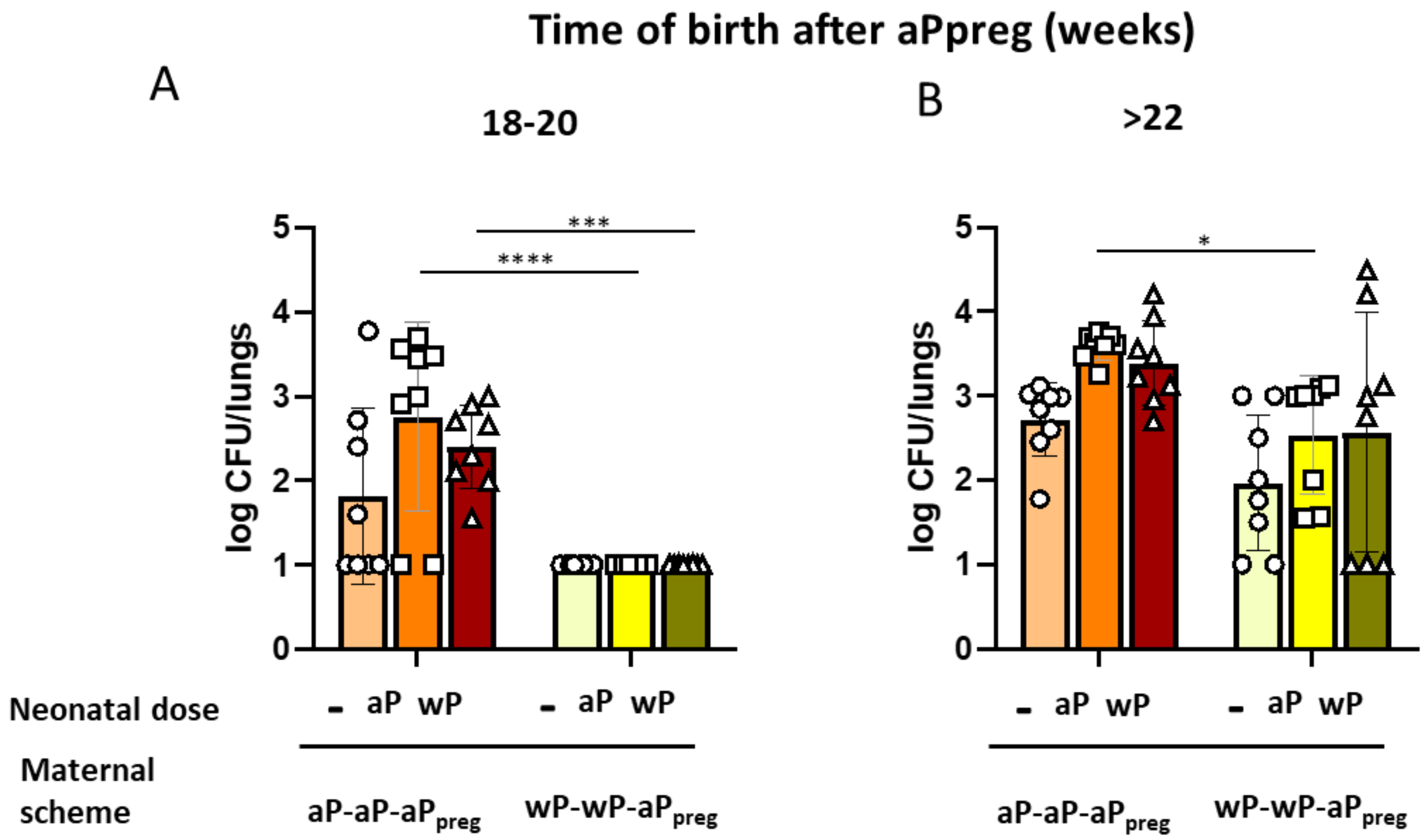

Time of birth after aPpreg (weeks)
A
18-20
B
>22
log CFU/lungs
0
1
2
3
4
5
***
****
*
Neonatal dose
- aP wP
- aP wP
- aP wP
- aP wP
Maternal scheme
aP-aP-aPpreg
wP-wP-aPpreg
aP-aP-aPpreg
wP-wP-aPpreg
